# Parasitic Twin Presenting Rudimentary Upper Limbs Causes a Unique Spectrum of Anomalies of Autosite

**DOI:** 10.4274/balkanmedj.2018.0781

**Published:** 2018-11-15

**Authors:** Ivana Kavecan, Milan Obrenovic, Jadranka Jovanovic Privrodski, Boris Privrodski, Mihajlo Jeckovic

**Affiliations:** 1 University of Novi Sad, Faculty of Medicine, Hajduk Veljkova, Novi Sad, Serbia; 2 Institute for Children and Youth Health Care of Vojvodina, Hajduk Veljkova, Novi Sad, Serbia

A female newborn infant was born with a dorsal mass on her cervicothoracic region (Figure 1a). The mass consisted of accessory partial and hypoplastic upper limbs (arms, three fingers, of which one came off, a hypoplastic ulna, radius, humerus, and two scapulas) completely covered with skin. The dorsal mass measured approximately 10 cm in diameter, was soft, showed no movement, was sensitive to touch, and was associated with a spectrum of other anomalies (Figure 1b). The proband was the third child of non-consanguineous parents. Two children from previous pregnancies were healthy. The family history was unremarkable. Further examinations such as three-dimensional computed tomography showed specific skeletal anomalies (Figure 1c), whereas magnetic resonance imaging showed multiple anomalies, including meningomyelocele, ventriculomegaly, the deformation of the vertebra and ribs, and a Bochdalek hernia. Based on these observations, the diagnosis was established as a heteropagus conjoined twin (Figure 1d). Chromosome analysis revealed a normal female karyotype, 46,XX. Written informed consent was obtained from patient's parents.

Conjoined twins with or without the associated congenital anomalies occur infrequently, with an estimated incidence of 1 in 50.000 to 1 in 100.000 live births (1). A heteropagus conjoined twin is not viable because it is a partially formed parasitic monozygotic twin, asymmetrically attached, and completely dependent for survival on the viable and dominant twin, termed as the autosite. A conjoined twin joined dorsally along the vertebral column is known as a “parasitic rachipagus,” from the Greek words “rachi” (spine) and “pagus” (fixed), and is the rarest type (2). The etiology of parasitic conjoined twins remains uncertain. It has been suggested that the incomplete division of the blastocyst around the second week of gestation results in conjoining. One twin dies, but some parts of the body continue to grow and remain attached to the dominant twin, which prevents closure of the neural tube during development and results in various anomalies in the living twin (3). For all types of conjoined twins, an appropriate clinical and imaging evaluation, including computed tomography, ultrasound, and magnetic resonance imaging, is necessary for differential diagnosis and to assess the potential for future developmental anomalies. Surgical excision is the definitive therapy; however, in this case, the mother did not accept surgical treatment because of the surgical and postsurgical risks. The patient’s outcome and prognosis depend on the extent of conjoined sharing, localization of the fusion, and associated anomalies. The majority of reported parasitic twins present with dysraphic spinal columns and various cord anomalies, many of which are unique. However, the same spectrum of anomalies described for this patient has not been reported previously. In addition, the purpose of this report is to emphasize the importance of imaging diagnosis in the multidisciplinary approach of multiple congenital anomalies.

## Figures and Tables

**Figure 1:**
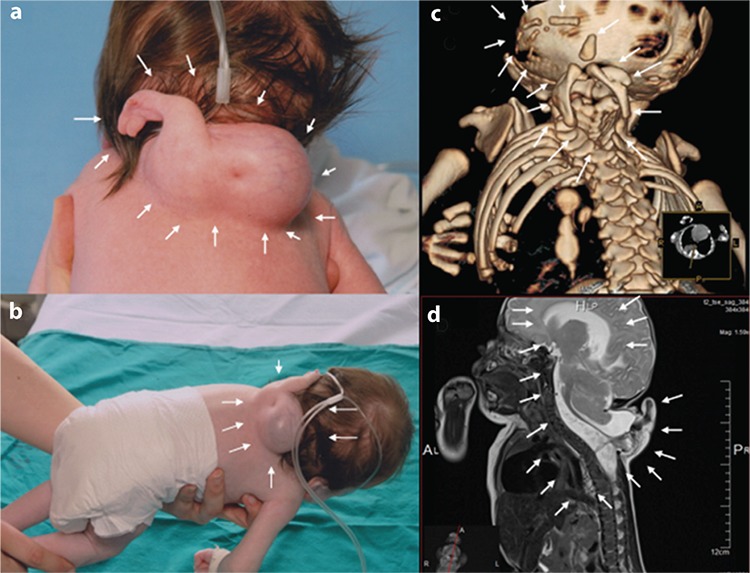
a-d. Meningomyelocele of the autosite and the rudimentary accessory upper limbs of the “parasitic” twin attached to the autosite (a, b), 3D-computed tomography revealing the fusion location of the accessory limbs of the “parasitic” twin with the autosite. There are two hypoplastic scapulae of the parasitic twin behind Th7-9, one hypoplastic humerus and ulna, one hypoplastic carpal bone, and five hypoplastic phalangeal bones of the two digits. In complete fusion of the posterior arches of the cervical and thoracic vertebrae (c), magnetic resonance imaging revealing eccentric meningomyelocele, ventriculomegaly, simplified gyral patterning, the deformation of the spinal cord along C1-Th12, and a Bochdalek hernia (d).
